# How can National Government Policies Improve Food Environments in the Netherlands?

**DOI:** 10.3389/ijph.2022.1604115

**Published:** 2022-03-07

**Authors:** Sanne K. Djojosoeparto, Carlijn B. M. Kamphuis, Stefanie Vandevijvere, Maartje P. Poelman

**Affiliations:** ^1^ Department of Human Geography and Spatial Planning, Faculty of Geosciences, Utrecht University, Utrecht, Netherlands; ^2^ Department of Interdisciplinary Social Science, Faculty of Social and Behavioural Sciences, Utrecht University, Utrecht, Netherlands; ^3^ Sciensano, Brussels, Belgium; ^4^ Chair Group Consumption and Healthy Lifestyles, Wageningen University & Research, Wageningen, Netherlands

**Keywords:** public health, government, obesity, overweight, food environment, policies, infrastructure support

## Abstract

**Objectives:** Government policies are essential to create food environments that support healthy diets. The aims of this study were 1) to benchmark the implementation of Dutch government policies influencing food environments, and 2) to identify and prioritize actions to improve food environments in the Netherlands.

**Methods:** The Healthy Food Environment Policy Index (Food-EPI) was applied. The Food-EPI includes 46 indicators of food environment policy and infrastructure support. Independent experts (n = 28) rated the extent of implementation on these indicators against international best practices, and formulated and prioritized policy and infrastructure support actions to improve food environments.

**Results:** Most policy indicators were rated as having a low (50%) or very low (41%) level of implementation. Most infrastructure support indicators were rated as having a fair (42%) or medium (42%) level of implementation. 18 policy and 11 infrastructure support actions were recommended by experts to improve food environments in the Netherlands.

**Conclusion:** There is large potential for the Dutch national government to strengthen its policy action and infrastructure support in order to improve the healthiness of food environments in the Netherlands.

## Introduction

Overweight, obesity and diet-related chronic diseases are a major public health challenge globally [1, 2]. In the Netherlands, approximately 50% of the adult population is overweight [3, 4]. An unhealthy diet is an important determinant of overweight, obesity and diet-related chronic diseases [5, 6]. Unhealthy diets are not merely the result of individual decisions, but strongly influenced by the food environment [7–9].

The food environment can be defined as the physical (e.g., food availability, marketing), economic (food prices), policy and sociocultural surroundings, opportunities and conditions that influence people’s food choices and nutritional status [9]. Over the past few decades, the availability and marketing of ultra-processed, high-fat and sugar-rich products increased, and prices of these products have decreased relatively to healthier foods [8–10]. As commercial interests have been allowed to prevail over public health, this has resulted in so-called obesogenic environments, in which unhealthy food choices are easier made than healthy food choices [8, 11–13].

To correct for this market failure, it is essential that governments develop policies to reverse the obesogenic nature of food environments [8, 11, 14]. Structural, government policies can play an important role to create healthy food environments, supporting the entire population to make healthy food choices [14–17]. These policies are known to be more effective in improving population diets than interventions which address individual behaviour (e.g., health mass media campaigns) [8, 18]. Globally, the implementation of policies to create supportive food environments is low [8, 19]. However, some governments are making progress, for example the Chilean government which implemented a Law of Food Labelling and Advertising, to introduce easy-to-understand front-of-pack labelling and specific messages addressing critical nutrients and to restrict unhealthy food marketing to children across media [20, 21].

In the Netherlands, Article 22 of the Dutch Constitution states that the government should take measures to promote public health [22]. The Dutch government has indeed implemented several voluntary measures to create healthy food environments. For instance, in 2014, the Dutch government signed an Agreement on Product Improvement with the food industry to reduce the amounts of salt, saturated fat and added sugar in products [23]. More recently, in 2018, the Dutch government signed the “National Prevention Agreement” (NPA) together with more than seventy public and private organizations [24, 25]. The NPA specifies goals to reduce overweight among adults from 48.7% in 2017 to 38% in 2040, and among children and adolescents from 13.5% in 2017 to 9.1% in 2040. In addition, the NPA aims to reduce obesity among adults from 14.5% in 2017 to 7.1% in 2040, and among children and adolescents from 2.8% in 2017 to 2.3% in 2040 [24, 25]. To achieve these goals, several voluntary actions have been described in the NPA, e.g., supermarkets will encourage consumers to buy products that are in line with Dutch dietary guidelines (Wheel of Five); the government will introduce a new, broadly supported food-choice logo; and a restriction on the use of licensed media characters aimed at children under 13 years of age on product packaging and point-of-sale materials will be included in the self-regulated Advertising Code for Food [24, 25]. While these voluntary actions can be supportive of healthy food environments, there is lack of structural policies in the NPA (such as the highly contested sugar-sweetened beverages tax, still not implemented in the Netherlands [26]). Contrary to these NPA actions, the Dutch government increased the value-added tax on all foods, including fruits and vegetables, from 6 to 9% in 2019 [27].

Although some actions regarding the improvement of food environments can be observed, a clear and comprehensive picture and evaluation of the current food environment policy landscape in the Netherlands is lacking. To gain more insight into where the largest policy implementation gaps lie and how the Dutch national government could improve its food environment policies, this study applied the Healthy Food Environment Policy Index (Food-EPI) developed by the International Network for Food and Obesity/non-communicable diseases Research, Monitoring and Action Support (INFORMAS) [14]. In applying the Food-EPI tool, this study aims:

1) To benchmark, against international best practices, the extent to which the Dutch national government has implemented policies contributing to a healthy food environment, as well as infrastructure support that facilitates effective policy development and implementation, and
2) To identify and prioritize context-specific actions that can improve food environments in the Netherlands.

## Methods

### Study Design

This mixed-methods study is conducted as part of the Policy Evaluation Network (PEN) (https://www.jpi-pen.eu/), and under the umbrella of INFORMAS (informas.org). Over the period 2019–2020, we adapted and applied the Food-EPI in the Netherlands [14]. Globally, the Food-EPI has already been applied in more than thirty countries [28]. All procedures performed were in accordance with the ethical standards of the institutional committee [Science-Geosciences Ethics Review Board (SG-ERB), Utrecht University, Netherlands (ERB Review Geo L-19254)] and the Helsinki declaration. All study participants signed informed consent before participation.

### Study Procedure

The Food-EPI is an international standardized tool and process to identify important gaps in policies and infrastructure support, and to identify and prioritize future actions to improve food environments [14]. The tool comprises indicators across seven food environment *policy* domains (food composition, labelling, promotion, prices, provision, retail, trade, and investment) and six *infrastructure support* domains (leadership, governance, monitoring and intelligence, funding and resources, platforms for interaction, health-in-all-policies) [14]. This study consisted of six steps (see Supplementary Material S1 for an overview of the steps and timeline), which are further outlined below.

#### Step 1: Tool Adaptation (February–May 2019)

Before applying the Food-EPI to the European context, PEN researchers consulted other researchers/experts to review the 47 original Food-EPI indicators. For each indicator, it was assessed whether the jurisdiction lies with the European Union, national governments or both. Furthermore, PEN researchers asked the participating researchers/experts to indicate whether indicators were clear, needed to be (dis)aggregated or whether indicators were missing. In the food promotion domain one indicator was disaggregated (into restricting promotion through online and social media and promotion in “non-broadcast” media) and one indicator was added (restricting promotion on food packages). In the food provision domain one indicator on public procurement standards in public sector settings was added. It was decided to include the trade domain (including two indicators) in the EU Food-EPI, but not in the national Food-EPI’s. This resulted in a total of 46 indicators included in the Dutch Food-EPI, i.e., 22 policy and 24 infrastructure support indicators (Supplementary Material S2).

#### Steps 2–3: “Evidence Document” and Online Benchmarking Survey

In step 2, evidence for the implementation of policies for each of the 46 Food-EPI indicators (up until 22 April 2020) in the Netherlands was collected through systematically searching for and reading national policy documents. We used several main sources to search for the relevant policy documents, including the national government websites (e.g., https://wetten.overheid.nl, htttps://www.rivm.nl, https://www.voedingscentrum.nl, https://www.rijksoverheid.nl). Via these websites we found information and links to additional useful documents including the Agreement on Product Improvement, the NPA, and the Advertising Code for Food. All policies identified at the national level with a potential influence on the food environment have been summarized in an 34-page “evidence document” [29]. This document was verified for completeness and accuracy by governmental officials, for example by officials working at the Ministry of Health, Netherlands Nutrition Centre and the National Institute for Public Health and the Environment.

After a brainstorm with the research team we developed a long list of relevant Dutch organizations in the field of food and nutrition, public health, obesity, and/or diet-related chronic diseases, i.e., academia, health organizations, health professional associations, non-governmental organizations, and local governments. Further, we created a long list of names of people working at these organizations, and purposively invited them to participate in the Dutch Food-EPI expert panel (March–May 2020). To ensure that all relevant expertise would be represented in the expert panel, invited experts were asked to supply the research team with any names of other relevant experts that should be invited for the Food-EPI expert panel.

In total 52 independent experts were invited. In step 3, they were asked to benchmark the implementation of policies and infrastructure support against international best practices during an online survey. A total of 28 experts filled out the survey (May–July 2020), of which 25 experts fully completed and 3 partly. Participants benchmarked the implementation of each of the 22 policy and 24 infrastructure support indicators, by comparing the level of implementation as described in the evidence document to international best practices (i.e., comprehensive examples of policy implementation worldwide which were provided for each indicator). The guidance that was given to the experts to determine the level of implementation has been included in Supplementary Material S3. A five-point Likert scale was included to benchmark the implementation of policies, with 1 = 0–20% implementation (=very low), 2 = 20–40% implementation (=low), 3 = 40–60% implementation (=medium), 4 = 60–80% implementation (=fair), and 5 = 80–100% implementation (=high). There was also a ‘cannot rate’ option and experts could comment on their rating in a text box.

Moreover, experts were asked to write down concrete actions (for each policy and infrastructure domain) that they considered important in order to improve the healthiness of food environments in the Netherlands.

#### Steps 4–6: Identification and Prioritization of Actions to Improve Food Environments in the Netherlands

Due to the 2020 Covid-19 bans on travel and meetings, the next logical step in the Food-EPI process, i.e., a face-to-face workshop with the expert panel to discuss the proposed actions, was not possible. Therefore, a different approach than outlined in the Food-EPI protocol [30] was taken, as described below in step 4–6.

#### Step 4 Online Workshops

To combine and narrow down (e.g., omit duplications) the actions as proposed by the expert panel (n = 28) during the online benchmarking survey (step 3), two online workshops of 3-hours each were held (September 2020). As there were many (189) actions formulated during the online benchmarking survey which had to be combined and narrowed down, we invited a selected group of experts (n = 4) (who also had completed the online benchmarking survey) to ensure an effective and efficient online discussion. Two of these experts were specialized in public health and nutrition working in health organizations and two of these experts were specialized in nutrition and food law/politics working in academia. For each domain, the experts were also consulted if any important actions were missing on the list.

#### Step 5a Refining Actions

The research team made final adjustments to the list of actions according to the input received during the workshops. This adjusted list of actions was then sent to the four experts who participated in the online workshops for verification.

#### Step 5b Online Selection Survey to Investigate Which Actions to Recommend

The expert panel (n = 28) was invited for a second online selection survey in October 2020. They were asked to indicate for each of the actions if they would recommend the Dutch government to implement this action, using a five-point Likert scale: 1) very much disagree 2) disagree 3) neutral 4) agree 5) very much agree. A total of 17 experts participated in this survey.

#### Step 6 Prioritization of the Recommended Actions

In the third and final online survey (November 2020), the expert panel (n = 28) was asked to prioritize the recommended actions that received an average score of 4.0 or higher in step 5b. A total of 21 experts completed this prioritization survey. Experts ranked the policy actions three times on 1) importance, 2) achievability and 3) equity, i.e., the effect on socioeconomic inequalities in diet. Experts ranked the infrastructure support actions twice on 1) importance and 2) achievability. Importance includes criteria on need, impact, and other positive and negative effects. Achievability includes criteria on feasibility, acceptability, affordability, and efficiency. And equity includes criteria on socioeconomic effects (regressive/progressive) and the extent to which a given policy requires environmental change rather than individual choices. Supplementary Material S4 includes a comprehensive description of the ranking criteria. When an action was ranked as #1 it was considered to be most important, achievable or equitable.

### Data Analysis

The mean score on the five point Likert scale was calculated for each indicator to determine the implementation of policies. The Gwet AC2 inter-rater reliability coefficient and its variance were determined using AgreeStat software (Agreestat 2015.6.1, Advanced Analytics, Gaithersburg, United States). For estimation of the variance, the sample of subjects to rate was set at 100% since all indicators of the Food-EPI were included for rating, while the sample of raters was set at 54% (as per the response rate of experts invited), and the finite population correction was applied (step 3).

Regarding step 5b, the mean score was calculated for each action based on the five point Likert scale. Actions with a mean score of 4.0 or higher were included in step 6.

In step 6, we identified the highest prioritized policy and infrastructure actions by summing the ranking scores for each action. First, we calculated the scores for importance and achievability separately. Second, we calculated the total score for each action by summing the scores on importance and achievability. Sum scores could vary from 42 to 756 (policy domains) and from 42 to 462 (infrastructure support domains). A lower sum score indicated a higher perceived priority. These sum scores were used to determine the top 5 prioritized policy actions and the top 5 prioritized infrastructure support actions. For the policy actions, we also calculated the sum of the scores on equity for each action and determined the top 5 actions which were perceived most effective to reduce socioeconomic inequalities in diet.

## Results

### Expert Panel

The 28 experts that participated in this study were working in academia, health organizations/health professional associations, non-governmental organizations (NGO’s) and local governments, and specialised in food, nutrition, public health, obesity and/or diet-related chronic diseases (Supplementary Material S5). In the online benchmarking survey participation was highest (12 experts from academia, six from health organizations/health professional associations, four from NGO’s and six from local governments), followed by the prioritisation survey (10 experts from academia, five from health organizations/health professional associations, three from NGO’s and three from local governments). The least experts participated in the selection survey (eight from academia, five from health organizations/health professional associations, three from NGO’s and one from a local government).

### Ratings of the Extent of Implementation of Policies and Infrastructure Support Influencing Food Environments Compared to Best Practice

**FIGURE 1:**
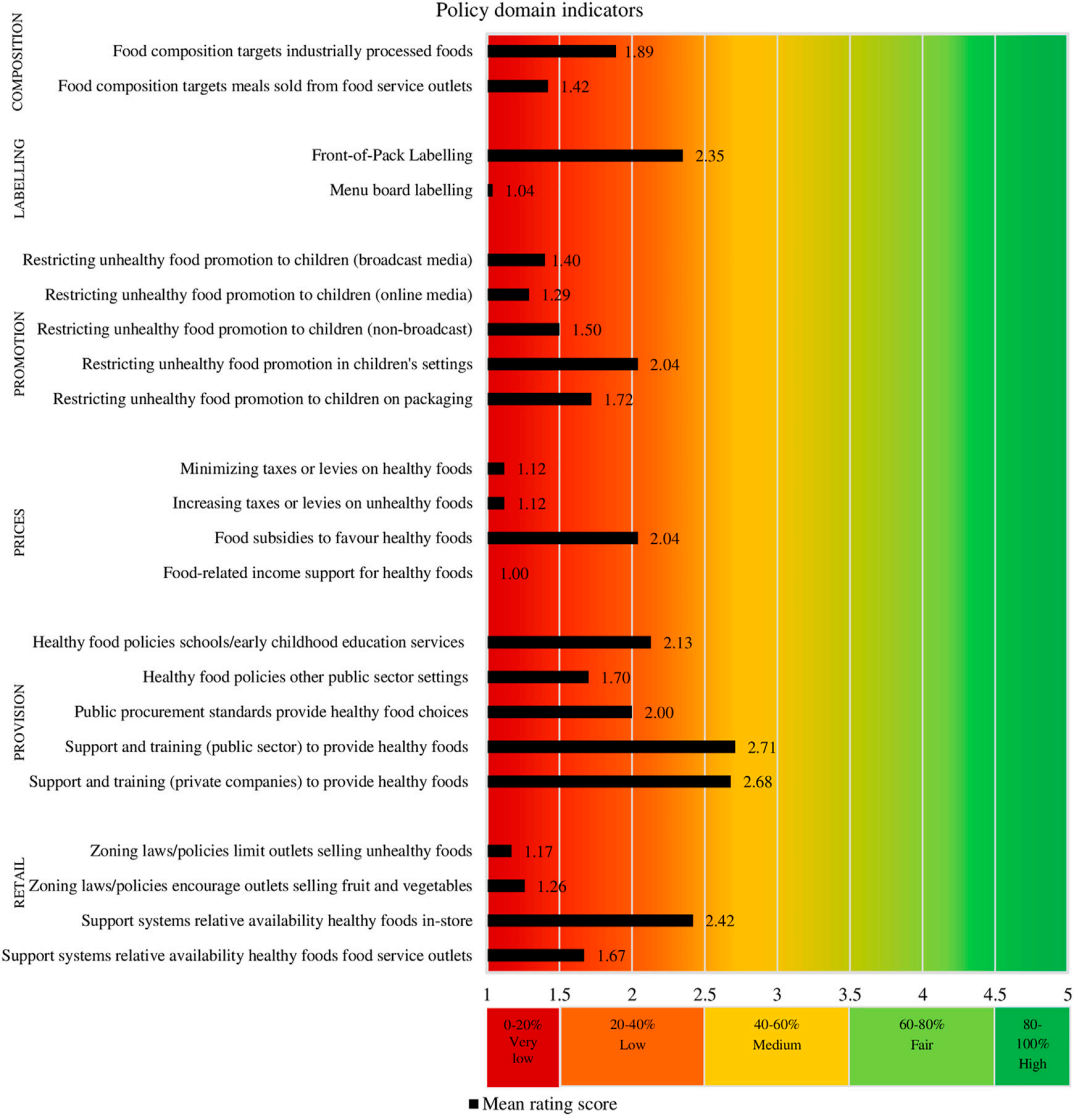
Ratings of the extent of implementation of policies influencing food environments. (Food-EPI study, the Netherlands, 2019–2020).

**FIGURE 2:**
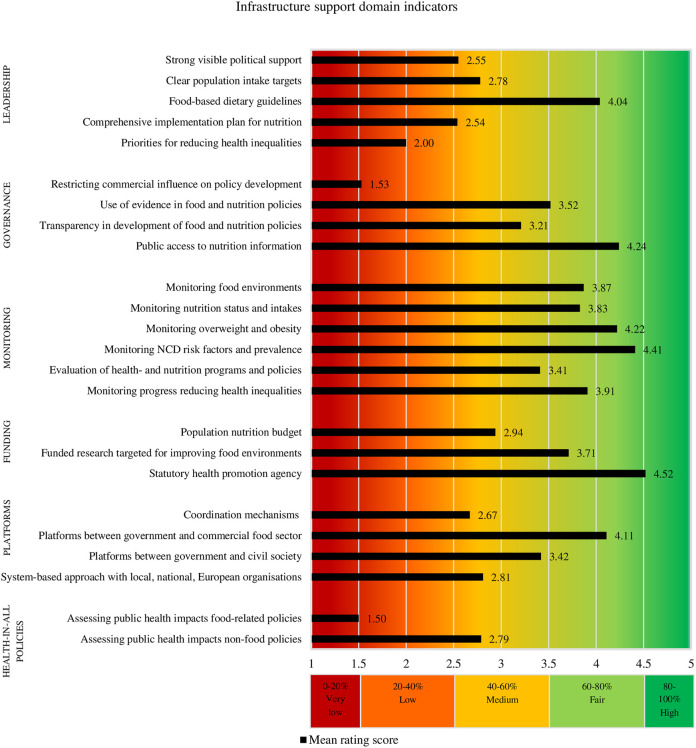
Ratings of the extent of implementation of infrastructure support influencing food environments. (Food-EPI study, the Netherlands, 2019–2020).

Figures 1 and 2 present for each Food-EPI indicator separately, the mean implementation score of policies and infrastructure support in the Netherlands compared to international best practices, according to the experts. The Inter-rater reliability (Gwet’s AC2) for all Food-EPI indicators was 0.57 (95% CI = 0.51–0.62), which indicates that there was moderate agreement among experts about the implementation of policies against international best practices. There was strong agreement about the policy indicators (Gwet’s AC2 was 0.78; 95% CI = 0.73–0.83), but lower agreement about the infrastructure support indicators (Gwet’s AC2 was 0.46; 95% CI = 0.39–0.53).

#### Policy Domains

The implementation of 50% of the indicators in the policy domains (11 of the 22 indicators) was rated as being “low” (20–40% implementation) (Figure 1). Yet, the implementation of 41% of the policies (nine of the 22 indicators) was rated even being “very low” (0–20% implementation). The expert panel considered the implementation of policies with respect to two of the 22 (9%) policy indicators as being “medium” (40–60% implementation).

#### Infrastructure Support Domains

The implementation of infrastructure support indicators was generally rated higher than policy indicators (Figure 2). The implementation of one of the 24 infrastructure support indicators, namely “having a statutory health promotion agency in place” (*Funding domain*) was rated “high” by the expert panel. This includes the Netherlands Nutrition Centre and the National Institute for Public Health and the Environment.

Further, the implementation of 10 of the 24 infrastructure support indicators (42%) was rated being “fair,” another 10 (42%) as being “medium,” and three indicators (12%) were rated as having “low” implementation compared to international best practices (Figure 2). In contrast to the policy indicators, no infrastructure support indicators were rated as having “very low” implementation.

### Identification and Prioritization of Actions to Improve Policies and Infrastructure Support

Based on step 3 (benchmark survey), step 4 (workshops) and step 5a (refinements), a total of 46 actions were proposed by the expert panel, namely 27 policy actions and 19 infrastructure support actions. In step 5b (selection survey), a total of 29 actions, including 18 policy actions and 11 infrastructure support actions were scored with a 4.0 or higher and thereby recommended to the national government to create healthy food environments in the Netherlands.

#### Recommended and Prioritized Policy Actions

The 18 policy actions recommended by the experts are detailed in Table 1. The actions are listed in order of priority considering both importance and achievability. The five actions with the highest potential to reduce dietary socioeconomic inequalities according to the experts are marked with an asterisk (*).

**TABLE 1:** Policy actions to create healthy food environments, recommended by the Food-EPI expert Panel (listed in order of prioritization on a combination of importance and achievability). (Food-EPI study, the Netherlands, 2019–2020).

Ranking	Sum score importance + achievability	Domain	Action
1	193	Food composition*	Ensure that the new product improvement system, in continuation of the agreement on product composition improvement, meets at least the following requirements
			- It includes more ambitious food composition targets than the current targets in the agreement on product composition improvement
			- It includes annual targets to reduce the amounts of salt, saturated fat and added sugars in all product categories which have an impact on the salt, saturated fat, and added sugars intake, where a reduction in one nutrient does not lead to an increase in another nutrient
			- There is a clear timeline with annual independent monitoring including baseline measurement, with publicly accessible reporting, to make the progress visible
			- It includes proven effective incentives per product category that ensure that food producers comply with agreements
2	275	Food promotion*	Ban all forms of marketing (Article 1 of the Dutch Advertising Code) aimed at children under the age of 18 years old for foods that fall outside the Dutch healthy dietary guidelines (i.e., the Wheel of Five) (an advertisement is aimed at children when the advertisement reaches an audience consisting of 10% children under 18 or more), via
			- media channels such as TV, radio, online and social media, point of sale, packages, games, cinema, print, sponsorship, kids clubs, sales promotion, product placement, films, peer-to-peer etc.
			- marketing methods such as the use of children’s idols, cartoons, animation figures, games, puzzles etc.
3	276	Food prices*	Increase the prices of unhealthy foods such as sugar-sweetened beverages, for example via a proven effective VAT-increase or excise tax
4	306	Food provision/ retail	Formulate clear rules and regulations for caterers, quick service restaurants, supermarkets and shops to increase the relative availability of healthy foods (with sufficient fiber, vitamins, and/or minerals) compared to the total food product availability
5	315	Food prices*	Decrease the prices of healthy foods such as fruit and vegetables, for example by reducing the VAT to 0% (when this is possible with the new European legislation)
6	335	Food retail/ food promotion	Encourage supermarkets and food producers to promote healthy foods via proven effective incentives
7	352	Food promotion	Ensure that supermarkets and food producers report annually in a measurable and comparable manner about actions, promotions and advertising aimed at healthy foods in relation to the total product promotion
8	360	Food composition	Encourage the European Union to remove bottlenecks so that the Netherlands can make binding agreements with food producers to achieve product improvement targets, including sanctions imposed by the government in the event of non-compliance
9	381	Food composition	Initiate an agreement to improve meal composition for caterers as well as quick service restaurants with targets to reduce the amounts of salt, saturated fat and added sugars and increase the amounts of fiber, vitamins and minerals (through healthy foods) in meals sold by caterers and quick service restaurants, for example by including gradual targets in such an agreement
10	434	Food prices*	Finance food-related income support, for example by providing vouchers to people below a certain income level to purchase healthy foods free of charge (such as fruits and vegetables, such as the Healthy Start programma in the UK)
11	448	Food retail	Formulate clear rules and regulations for retail, catering and hospitality, to discourage unhealthy food choices in supermarkets, shops, canteens and quick service restaurants and encourage healthy food choices, for example banning sweets at the checkout counter or prescribing a maximum percentage of unhealthy foods in relation to the total food availability and in promotions
12	465	Food provision	Facilitate the provision of healthy foods and school meals (e.g., lunch) in primary schools by providing an infrastructure (staffing, logistics, procurement), policies and subsidies (and make the contribution of parent income-related, whereby the school meals (e.g., lunch) are free for lower socioeconomic groups)
13	474	Food provision	Tighten the criteria of the dietary guidelines “Healthier Canteens” and “Healthier Eating environments” of the Dutch Nutrition Centre and encourage schools, hospitals, company canteens, and government-funded institutions to implement these guidelines with proven effective incentives to ensure compliance
14	489	Food prices	Invest the revenues of the increased prices on unhealthy foods (VAT, excise tax) in broad proven effective health programs for promoting healthy food consumption and prevention of lifestyle-related (chronic) diseases (e.g., promotion of healthy foods, subsidy for providing healthy foods at schools)
15	495	Food retail	Implement regulations with regard to improving the food availability in municipalities, for example by providing local governments certain criteria which prohibit the presence of fast food outlets or quick service restaurants or set a maximum number of such food providers (“zoning”)
16	527	Food promotion	Ban sponsorship by food producers who have unhealthy foods in their product portfolio and ban sponsoring of unhealthy foods in schools, hospitals, company canteens, government-funded institutions, sport canteens (e.g., sponsored soft drinks vending machines in these locations)
17	528	Food prices	Implement a ‘True Pricing’ policy, in which, among other things, the health care costs arising from health problems related to the consumption of unhealthy foods, are passed on in the price of these products (making healthy foods cheaper and unhealthy foods more expensive)
18	529	Food provision	Facilitate the provision of healthy foods in secondary schools by providing an infrastructure (staffing, logistics, procurement), policies and subsidies for the provision of healthy school meals, a healthy lunch assortment and healthy products in vending machines

*The top 5 prioritized actions on equity are marked with an Asterisk (*).

Four of the top 5 prioritized policy actions on importance and achievability, also appeared in the top 5 actions with the greatest potential to reduce dietary socioeconomic inequalities. These four actions, together with the other top 5 action on importance and achievability, and the other top 5 action on equity (six in total) were recommended to the government for immediate implementation.

**FIGURE 3:**
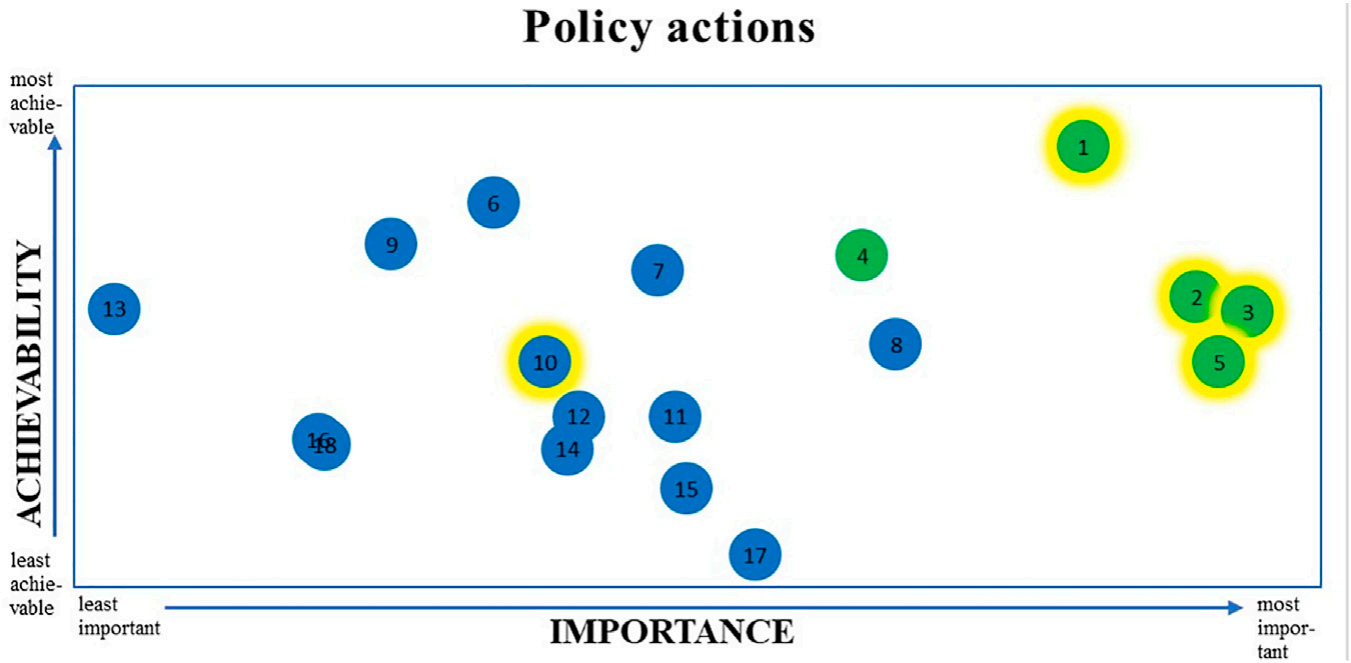
Importance and achievability of recommended policy actions for the Dutch national government and the top 5 actions which have the greatest potential to reduce socioeconomic inequalities in diet*. *The top 5 priority policy actions on a combination of importance and achievability are shown in green; the five actions which have the greatest potential to reduce socioeconomic inequalities in diet are indicated by the yellow shadow. See Table 1 for a description of the 18 policy actions. (Food-EPI study, the Netherlands, 2019–2020).

In Figure 3, the scores on importance and achievability for each action are plotted in a graph, and the five actions with the greatest potential to reduce socioeconomic inequalities in diet are indicated by a yellow shadow.

#### Recommended and Prioritized Infrastructure Support Actions

**FIGURE 4:**
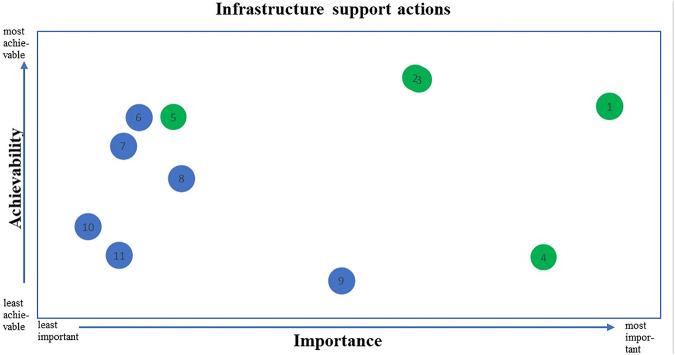
Importance and achievability of recommended infrastructure support actions for the Dutch national government*. *The top 5 priority infrastructure support actions on a combination of importance and achievability are shown in green. See Table 2 for a description of the 11 infrastructure support actions. (Food-EPI study, the Netherlands, 2019–2020).

The 11 infrastructure support actions recommended by the Food-EPI expert panel are detailed in Table 2. The actions are listed in order of priority considering both importance and achievability. The top 5 prioritized actions were recommended to the government for immediate implementation. Each infrastructure support action is plotted on importance and achievability in Figure 4.

**TABLE 2:** Infrastructure support actions, recommended by the Food-EPI expert panel (listed in order of prioritization on a combination of importance and achievability).

Ranking	Sum score importance + achievability	Domain	Action
1	135	Leadership	Develop a government-wide national prevention policy and implementation plan containing universal, selective, indicated and care-related prevention measures, aimed at, among other things, a healthy food consumption and the reduction of diet-related (chronic) diseases among the entire population. Address the physical, socioeconomic and digital living environment so that it contributes to the promotion of health and underlying socioeconomic determinants of unhealthy food consumption (e.g., poverty, stress). Make all ministries co-owners of this policy and encourage the collaboration between the ministries in this field
2	169	Platforms for interaction	Support local governments with developing and implementing prevention measures aimed at a healthy food consumption, a healthy food environment and the reduction of diet-related (chronic) diseases
3	169	Monitoring and intelligence/governance	Develop concrete, measurable targets with regard to prevention measures (preferably integrated in a national prevention policy), aimed at a healthy food consumption, a healthy food environment and the reduction of diet-related (chronic) diseases, which can be tested by an independent organization (RIVM) and make the total overview of the achieved and not achieved results on these targets publicly available
4	236	Funding and resources	Increase the budget for universal, selective, indicated and care-related prevention in the national budget, with at least 10% of the health care budget going to prevention in the first 4 years and gradually reversing the financing pyramid for health care (with the vast majority of it going to prevention instead of curative care)
5	253	Monitoring and intelligence	Develop an instrument for reporting about the food availability in supermarkets, shops, quick service restaurants and catering that shows the share of healthy foods in relation to the total food product range, and make binding agreements with the involved parties (local governments, schools, hospitals, food producers etc.) about monitoring and reporting thereof
6	262	Governance	Ensure transparency about the decision-making of prevention measures (preferably integrated in a national prevention policy) aimed at a healthy food consumption, a healthy food environment and the reduction of diet-related (chronic) diseases, by reporting about the process and taken decisions and making these publicly available
7	282	Funding and resources	Develop a joint knowledge agenda and a comprehensive research program for institutions and science [National Institute for Public Health and Environment (RIVM), Local Public Health Services (GGD-en), Netherlands Organisation for Health Research and Development (ZonMw), Dutch Research Council (NWO)], including funding for the evaluation of existing government policies and the development of a new, structural policy, aimed at upstream factors 1) to promote the availability of healthy foods, 2) to reduce overweight, obesity and diet-related diseases and 3) to utilize the health potential
8	285	Governance	Develop a framework with binding agreements about the involvement of and cooperation with non-state actors^1^ in the development and implementation of prevention measures aimed at a healthy food consumption, a healthy food environment and the reduction of diet-related (chronic) diseases, as also described in the WHO Framework of Engagement with Non-State Actors (FENSA)^2^
9	301	Health-in-all-policies	Develop an intersectoral, health policy (health-in-all policies; including a healthier food system) with shared ambitions, concrete targets and multi-year plans and make this legally binding (by mentioning health explicitly in policy programs and integrating health into all ministerial budgets)
10	336	Monitoring and intelligence	Increase the control and enforcement by the Dutch Food and Consumer Food Safety Authority (NVWA) on food labels and health claims in addition to the control and enforcement that currently mainly focuses on allergens and food safety. Perform product measurements as part of this control and enforcement
11	344	Health-in-all-policies	Develop Health Impact Assessments (HIAs) that pay attention to the health of humans, animals and planet and that create clear frameworks for the various policy areas and sectors about what needs to be evaluated. Make HIA’s mandatory in the development of policies (for example in ex ante evaluations and to include health interests in the development of policies) and for sectors (as is done with Environmental Impact Reports)

1 Civil society organizations, private sector, philanthropic foundations and academic organizations.

2 https://www.who.int/about/collaborations/non-state-actors/A69_R10-FENSA-en.pdf?ua=1

(Food-EPI study, the Netherlands, 2019–2020).

## Discussion

This study showed that there are several gaps in the implementation of food environment policies and infrastructure support in the Netherlands. Moreover, results indicate that there are relatively more implementation gaps with regard to policies directly influencing food environments (*policy domains*) than with regard to infrastructure support facilitating the development and implementation of policies (*infrastructure support domains*). A total of 18 policy and 11 infrastructure support actions to create healthier food environments in the Netherlands have been identified that can be implemented by the Dutch government.

The outcomes of this Food-EPI study for the Netherlands were in line with international observations. An 11-country Food-EPI comparison study showed that the implementation of infrastructure support was rated higher than the implementation of food environment policies in all countries, except Chile [20]. Also in Ireland and Norway, where comparable Food-EPI studies were conducted as part of the Policy Evaluation Network, the implementation of infrastructure support was rated higher than the implementation of the policy indicators [31, 32].

There are a number of possible explanations for the low implementation of policies directly influencing food environments. First, the food industry has a diverse range of strategies to influence governmental policies, such as lobbying, participation in meetings with governments, and promoting industry-preferred solutions such as education and voluntary initiatives which rely on self-regulation, rather than mandatory governmental regulations [12, 33–37]. Second, the influence of these strategies is strengthened by a lack of political will to implement structural, universal, obesity and diet-related chronic diseases prevention measures [37]. The WHO indicated that not one single country has managed to turn around the obesity epidemic, because of a failure of political will to take on big business [38]. Like the default in many countries [39], voluntary self-regulation is the common approach to improve food availability and promotion in the Netherlands. The past governing coalitions consisted of mainly liberal and confessional parties where self-regulation by the industry has been an important tradition in health policy development and implementation [40]. To illustrate, in 2014, the Minister of Health came to a national agreement with representatives of the food industry to improve product composition [23, 41]. Businesses concluded voluntary chain agreements to reduce the content of salt, saturated fat and added sugar in their products [42]. There were no incentives from the government if the industry would not meet these agreements [23]. More recently (2018), as part of the NPA, the national government installed a committee with 70 organizations that represented a wide variety of stakeholders including associations of health charities, municipalities, primary and secondary education, municipal health services, health professionals, health care insurance companies, but also associations of the food industry, supermarkets, catering companies, and restaurants [43]. As part of this committee agreements to reduce overweight prevalence in the Netherlands where brokered [43]. This led to an NPA only containing voluntary measures to create healthy food environments [24, 25]. According to the National Institute for Public Health and the Environment, with the agreements to improve product composition only small steps are taken [44–46] and the measures in the NPA will only lead to a limited slowdown in the increase in overweight and obesity [47]. Indeed, according to literature, improvements of the food environment as a result of voluntary self-regulated approaches by the industry are mostly weak and there is little evidence of their effectiveness in improving population diets and preventing obesity and diet-related chronic diseases [39, 48].

As appears from the recommended and prioritized actions in our study, there is a need for less self-regulation and more ambitious, structural, universal interventions by the Dutch government. This need has also been recognized by the State Secretary for Health in a reaction to our Food-EPI report [49]. In the recently published coalition agreement 2021–2025, the new Dutch government announces a few structural and strict measures towards healthier food environments [50]. Actions included in this agreement are making *binding* agreements with the food industry about healthier foods, increasing taxes on sugar-sweetened beverages and investigating how to introduce a sugar tax and lower the current VAT tariff of 9% on vegetables and fruit to 0% [50]. Furthermore, the government promises to protect children against inappropriate online promotion and marketing [50]. However, it is not specified if this will also include protection against food marketing, which is currently regulated via the Advertising Code for Food products (2019) [51] initiated by the Dutch Food Industry Federation [52, 53].

Like the Netherlands, most European countries currently also have mainly voluntary initiatives [54], but some have already implemented more extensive measures. For example, regarding restricting unhealthy food marketing to children, the UK is considering a total ban on online advertising of foods high in fat, sugar, or salt to children [55]. In Portugal, Law 30/2019 restricts unhealthy food advertising directed to children via broad-cast media and digital marketing [56].

Related to price measures, various other European countries have already implemented food-related health taxes, such as the sugar-sweetened beverages taxes in the UK, Ireland, France, Spain, Portugal and the public health product tax in Hungary [57]. Also, several European countries apply a lower VAT-tariff on fruits and vegetables than the 9% in the Netherlands, such as the UK and Ireland (0%), Spain and Italy (4%) and Poland and Latvia (5%) [58]. Such structural policies more likely result in sustainable food consumption changes of the whole population including vulnerable groups, which could contribute to a reduction in socioeconomic inequalities in diet [8, 59, 60]. As the impact of combined interventions is greater than the impact of single interventions, experts in this study emphasized that measures should be part of a comprehensive, population-wide approach to prevent obesity and diet-related chronic diseases [61, 62].

Differently than in other Food-EPI studies, in the PEN Food-EPI’s experts were also asked to prioritize the policy actions on equity. Experts in our study indicated that price actions have the greatest potential to reduce socioeconomic inequalities in diet, which was also shown by an umbrella and systematic review [63, 64]. However, experts also indicated that food composition and marketing policies could be pro-equity, for which less empirical evidence was found [63, 64].

For this study we also have to consider that the Dutch national government is dependent on EU regulations. A Food-EPI study at EU-level was conducted to gain insight into the policies that need to be improved to create healthy food environments in EU Member States [65]. Thus, in addition to the actions that the Dutch national government can implement immediately, some actions (e.g., allowing a VAT of 0% on fruits and vegetables which was recently agreed on by the EU finance ministers and on which the European Parliament will be consulted [66]) cannot be implemented without policy changes at EU-level. It is therefore essential that national governments stimulate the EU to remove bottlenecks for creating healthy food environments at national level.

### Strengths and Limitations

This study has some important strengths. This is the first study in the Netherlands that applied a comprehensive mixed-methods approach in order to generate insight into the largest policy and infrastructure support implementation gaps as well as government actions to improve food environments. Second, policies described in the evidence document were verified by governmental officials and implementation of policies was evaluated by independent experts.

Nevertheless, some limitations should be acknowledged. First, due to the Covid-19 restrictions on travel and meetings, the workshop (step 4) was conducted online with a small group of experts instead of the envisaged face-to-face meeting with the entire expert panel. In addition, we were experiencing drop-out in participation, as a lower number experts participated in the follow-up surveys (n = 17, n = 21) compared to the first survey (n = 28), which showed the limitations of an online procedure. This might have impacted on the results regarding the recommended actions and ranking of the actions that should be considered. However, the diverse range of expertise of experts that did participate in the follow-up surveys, still make the results representative for the Dutch experts in the field of food, nutrition, public health, obesity, and/or diet-related chronic diseases. Moreover, compared to other international Food-EPI studies, the number of experts that participated in our final online prioritization survey (n = 21) is in line with other countries [31, 32]. Although we used an international standardized framework to assess food environmental policies from a public health perspective, the methodology is susceptible to subjectivity. A final limitation is that the Food-EPI does not identify *why* policies have or have not been successfully implemented [67]. Identifying the barriers and facilitators to implementing food environment policies could give important additional insights into how the national government could enable the implementation of these policies [68].

We also have some recommendations for future research. This study constructed scorecards (Figures 1, 2) on the implementation of national government policies, which facilitates monitoring of these policies over time, for example every five years. In the long-term, this study can contribute to a global database for monitoring and evaluating food environment policies. Another recommendation is to identify *why* recommended policies have or have not been successfully implemented, which can support uptake of policies [68]. A final recommendation is to compare the study outcomes, with outcomes of the other Food-EPI studies conducted as part of PEN (EU-level, Ireland, Norway, Poland, Germany) and the H2020 Science and Technology in childhood Obesity Policy (STOP) project (Slovenia, Spain, Portugal, Estonia, Finland).

### Conclusion

Experts consider the implementation of Dutch government policies directly influencing food environments largely as very low to low, while the implementation of infrastructure support was rated fair to medium. Recommended actions should be implemented by the Dutch government to create healthier food environments in the Netherlands.
