# Supplementary material for: How can National Government Policies Improve Food Environments in the Netherlands?
Source: Int J Public Health. 2022 Mar 7;67:1604115. doi: 10.3389/ijph.2022.1604115 (PMC8935556; doi:10.3389/ijph.2022.1604115)
Supplement: Supplementary file 1 [file DataSheet1.docx]

*International Journal of Public Health*

*Special Issue: Food as a Public Health Issue*

**SUPPLEMENTARY MATERIALS**

**How can national government policies improve food environments in the Netherlands?**

**Supplementary file 1**

**Figure S1 Steps of the Healthy Food Environment Policy Index 2019-2020 applied in this study (2019-2020) benchmarking the implementation of food environment policies and identifying priority actions for the Dutch national government.**

*[Food-EPI study, the Netherlands, 2019-2020]*

**Supplementary file 2**

This study applied the Healthy Food Environment Policy Index (Food-EPI), a monitoring framework, developed by the International Network for Food and Obesity/Non-communicable Diseases Research, Monitoring and Action Support (INFORMAS).^[[1]](#footnote-1)^

The Food-EPI includes seven policy domains that represent key aspects of food environments (food composition, labelling, marketing, provision, retail, prices and trade) that can be influenced by governments to facilitate the accessibility, availability, acceptability and affordability of foods contributing to a healthy diet. In addition, the Food-EPI is comprised of six infrastructure domains (leadership, governance, funding and resources, monitoring and intelligence, platforms for interaction and health-in-all-policies), which are based on the WHO building blocks for health systems, and facilitate policy development and implementation to create healthy food environments (Figure S2).

**Figure S2 The Healthy Food Environment Policy Index (Food-EPI)**

Swinburn B, Vandevijvere S, Kraak V, Sacks G, Snowdon W, Hawkes C, et al. (2013)

*[Food-EPI study, the Netherlands, 2019-2020]*


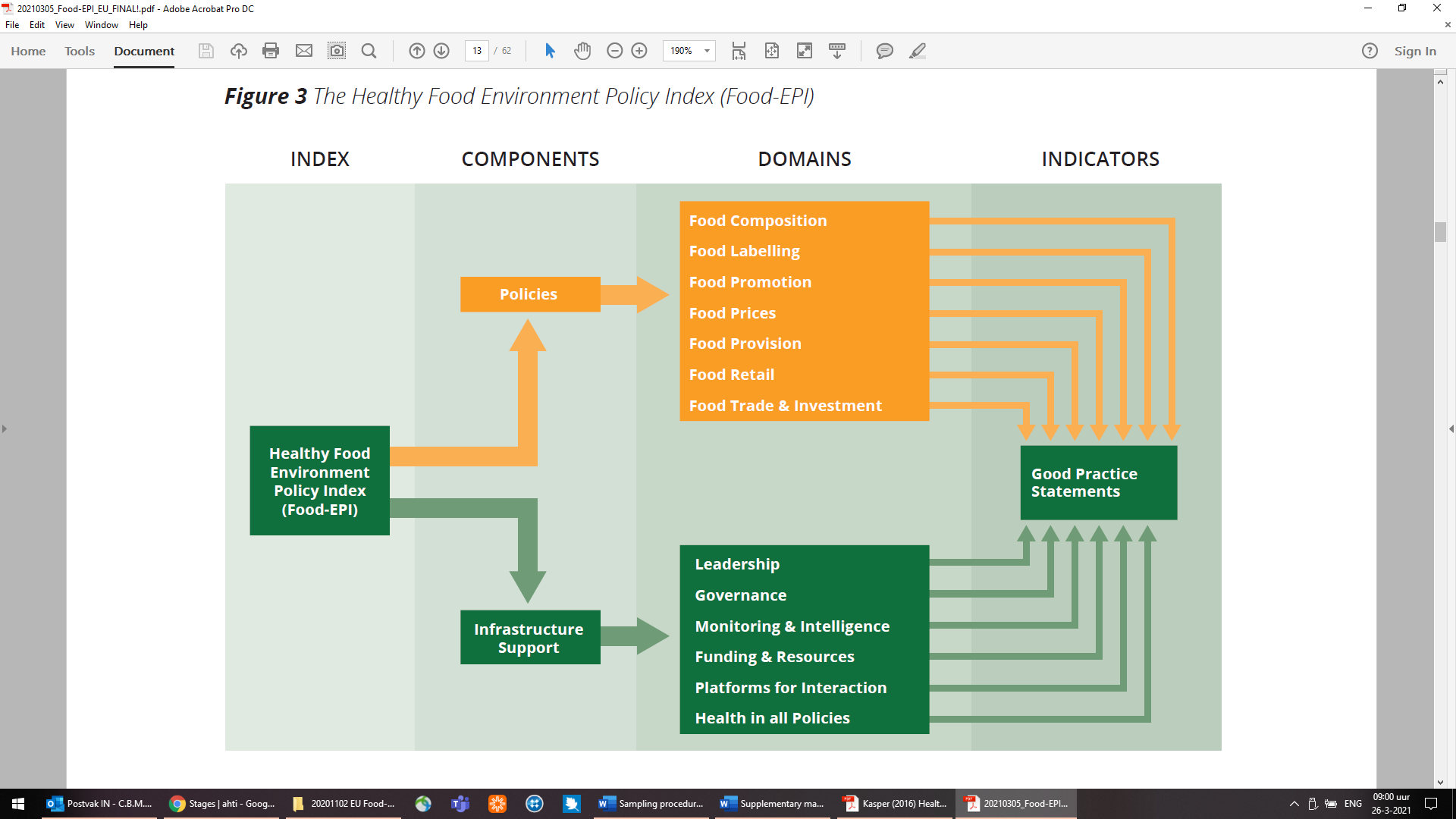


There are 50 good practice indicators contained in each of the domains that encompass actions necessary to improve the healthiness of food environments and to help prevent obesity and diet-related NCDs (see Table S1). 46 of these good practice indicators have been included in this Food-EPI study at national government level in the Netherlands (excluded are LABEL1, LABEL2, TRADE1 and TRADE2 as the jurisdiction of these indicators lies at the EU-level).

**Table S1 Food-EPI Domains and Indicators** *[Food-EPI study, the Netherlands, 2019-2020]*

| **Food-EPI Policy Domains** | |
| --- | --- |
| **Food-EPI Domain** | **Food-EPI Indicators** |
| DOMAIN 1 – FOOD COMPOSITION  Food composition targets/standards/restrictions for processed foods: There are government systems implemented to ensure that, where practicable, processed foods minimise the energy density and the nutrients of concern (salt, saturated fat, trans fat, added sugar). | **COMP1** Food composition targets/standards/restrictions have been established by the government for the content of the nutrients of concern (trans fats, added sugars, salt, saturated fat) in industrially processed foods, in particular for those food groups that are major contributors to population intakes of those nutrients of concern. |
|  | **COMP2** Food composition targets/standards/restrictions have been established by the government for the content of the nutrients of concern (trans fats, added sugars, salt, saturated fat) in meals sold from food service outlets, in particular for those food groups that are major contributors to population intakes of those nutrients of concern. |
| DOMAIN 2 – FOOD LABELLING  There is a regulatory system implemented by the government for consumer-oriented labelling on food packaging and menu boards in restaurants to enable consumers to easily make informed food choices and to prevent misleading claims**.** | **LABEL1** Ingredient lists and nutrient declarations in line with Codex recommendations are present on the labels of all packaged foods. |
|  | **LABEL2** Evidence-based regulations are in place for approving and/or reviewing claims on foods, so that consumers are protected against unsubstantiated and misleading nutrition and health claims. |
|  | **LABEL3** Evidence-based regulations are in place for approving and/or reviewing claims on foods, so that consumers are protected against unsubstantiated and misleading nutrition and health claims. |
|  | **LABEL4** A simple and clearly-visible system of labelling the menu boards of all quick service restaurants (i.e. fast food chains) is applied by the government, which allows consumers to interpret the nutrient quality and energy content of foods and meals on sale. |
| DOMAIN 3 – FOOD PROMOTION  There is a comprehensive policy implemented by the government to reduce the impact (exposure and power) of promotion of unhealthy foods to children across all media.  • Exposure of food marketing concerns the reach and frequency of a marketing message. This is dependent upon the media or channels which are used to market foods.  • The power of food marketing concerns the creative content of the marketing message. For example, using cartoons or celebrities enhances the power (or persuasiveness) of a marketing message because such strategies are attractive to children. | **PROMO1** Effective policies are implemented by the government to restrict exposure and power of promotion of unhealthy foods to children including adolescents through broadcast media (TV, radio). |
|  | **PROMO2** Effective policies are implemented by the government to restrict exposure and power of promotion of unhealthy foods to children including adolescents through online and social media. |
|  | **PROMO3** Effective policies are implemented by the government to restrict exposure and power of promotion of unhealthy foods to children including adolescents through nonbroadcast media other than packaging and online/social media. |
|  | **PROMO4** Effective policies are implemented by the government to ensure that unhealthy foods are not commercially promoted to children including adolescents in settings where children gather (e.g. preschools, schools, sport and cultural events). |
|  | **PROMO5** Effective policies are implemented by the government to ensure that unhealthy foods are not commercially promoted to children, including adolescents on food packages |
| DOMAIN 4 – FOOD PRICES  Food pricing policies (e.g., taxes and subsidies) are aligned with health outcomes by  helping to make the healthy eating choices the easier, cheaper choices. | **PRICES1** Taxes or levies on healthy foods are minimised to encourage healthy food choices (e.g. low or no sales tax, excise, value-added or import duties on fruit and vegetables). |
|  | **PRICES2** Taxes or levies on unhealthy foods (e.g. sugar-sweetened beverages, foods high in nutrients of concern) are in place and increase the retail prices of these foods by at least 10% to discourage unhealthy food choices, and these taxes are reinvested to improve population health. |
|  | **PRICES3** The intent of existing subsidies on foods, including infrastructure funding support (e.g. research and development, supporting markets or transport systems), is to favour healthy rather than unhealthy foods. |
|  | **PRICES4** The government ensures that food-related income support programs are for healthy foods. |
| DOMAIN 5 – FOOD PROVISION  The government ensures that there are healthy food service policies implemented in government-funded settings to ensure that food provision encourages healthy food choices, and the government actively encourages and supports private companies to implement similar. | **PROV1** The government ensures that there are clear, consistent policies (including nutrition standards) implemented in schools and early childhood education services for food service activities (canteens, food at events, fundraising, promotions, vending machines etc.) to provide and promote healthy food choices. |
|  | **PROV2** The government ensures that there are clear, consistent policies in other public sector settings for food service activities (canteens, food at events, fundraising, promotions, vending machines, etc.) to provide and promote healthy food choices. |
|  | **PROV3** The government ensures that there are clear, consistent public procurement standards in public sector settings for food service activities to provide and promote healthy food choices. |
|  | **PROV4** The Government ensures that there are good support and training systems to help schools and other public sector organisations and their caterers meet the healthy food service policies and guidelines |
|  | **PROV5** The Government actively encourages and supports private companies to provide and promote healthy foods and meals in their workplaces. |
| DOMAIN 6 – FOOD IN RETAIL  The government has the power to implement policies and programs to support the availability of healthy foods and limit the availability of unhealthy foods in communities (outlet density and locations) and in-store (product placement). | **RETAIL1** Zoning laws and policies are implemented to place limits on the density or placement of quick serve restaurants or other outlets selling mainly unhealthy foods in communities and/or access to these outlets (e.g. opening hours). |
|  | **RETAIL2** Zoning laws and policies are implemented to encourage the availability of outlets selling fresh fruit and vegetables and/or access to these outlets (e.g. opening hours, frequency i.e. for markets). |
|  | **RETAIL3** The Government ensures existing support systems are in place to encourage food stores to promote the in-store availability of healthy foods and to limit the in-store availability of unhealthy foods. |
|  | **RETAIL4** The government ensures existing support systems are in place to encourage the promotion and availability of healthy foods in food service outlets and to discourage the promotion and availability of unhealthy foods in food service outlets. |
| DOMAIN 7 – FOOD TRADE AND INVESTMENT  The government ensures that trade and investment agreements protect food sovereignty, favour healthy food environments, are linked with domestic health and agricultural policies in ways that are consistent with health objectives, and do not promote unhealthy food environments. | **TRADE1** The Government undertakes risk impact assessments before and during the negotiation of trade and investment agreements, to identify, evaluate and minimize the direct and indirect negative impacts of such agreements on population nutrition and health. |
|  | **TRADE2** The government adopts measures to manage investment and protect their regulatory capacity with respect to public health nutrition. |

| **Food-EPI Infrastructure Support Domains** | |
| --- | --- |
| **Food-EPI Domain** | **Food-EPI Indicators** |
| DOMAIN 8 – LEADERSHIP  The political leadership ensures that there is strong support for the vision, planning, communication, implementation and evaluation of policies and actions to create healthy food environments, improve population nutrition, and reduce diet-related inequalities. | **LEAD1** There is strong, visible, political support (at the head of government or state/ministerial level) for improving food environments, population nutrition, diet related NCDs and their related inequalities” |
|  | **LEAD2** Clear population intake targets have been established by the government for the nutrients of concern and/or relevant food groups to meet WHO and national recommended dietary intake levels. |
|  | **LEAD3** Clear, interpretive, evidenced-informed food based dietary guidelines have been established and implemented. |
|  | **LEAD4** There is a comprehensive, transparent, up-to-date implementation plan linked to national needs and priorities, to improve food environments, reduce the intake of the nutrients of concern to meet WHO and national recommended dietary intake levels, and reduce diet-related NCDs. |
|  | **LEAD5** Government priorities have been established to reduce inequalities or protect vulnerable populations in relation to diet, nutrition, obesity and NCDs. |
| DOMAIN 9 – GOVERNANCE  Governments have structures in place to ensure transparency and accountability, and encourage broad community participation and inclusion when formulating and implementing policies and actions to create healthy food environments, improve population nutrition, and reduce diet-related inequalities. | **GOVER1** There are procedures in place to restrict commercial influences on the development of policies related to food environments where they have conflicts of interest with improving population nutrition. For example: restricting lobbying influences. |
|  | **GOVER2** Policies and procedures are implemented for using evidence in the development of food and nutrition policies. |
|  | **GOVER3** Policies and procedures are implemented for ensuring transparency in the development of food and nutrition policies. |
|  | **GOVER4** The government ensures public access to comprehensive nutrition information and key documents (e.g. budget documents, annual performance reviews and health indicators) for the public. |
| DOMAIN 10 – MONITORING AND INTELLIGENCE  The government’s monitoring and intelligence systems (surveillance, evaluation, research and reporting) are comprehensive and regular enough to assess the status of food environments, population nutrition and diet-related NCDs and their inequalities, and to measure progress on achieving the goals of nutrition and health plans. | **MONIT1** Monitoring systems, implemented by the government, are in place to regularly monitor food environments (especially for food composition for nutrients of concern, food promotion to children, and nutritional quality of food in schools and other public sector settings), against codes/guidelines/standards/targets. |
|  | **MONIT2** There is regular monitoring of adult and childhood nutrition status and population intakes against specified intake targets or recommended daily intake levels. |
|  | **MONIT3** There is regular monitoring of adult and childhood overweight and obesity prevalence using anthropometric measurements. |
|  | **MONIT4** There is regular monitoring of the prevalence of NCD metabolic risk factors and occurrence. |
|  | **MONIT5** Major programs and policies are regularly evaluated to assess their effectiveness and contributions to achieving the goals of the nutrition and health plans. |
|  | **MONIT6** Progress towards reducing health inequalities or health impacts in vulnerable populations and social and economic determinants of health are regularly monitored. |
| DOMAIN 11 – FUNDING AND RESOURCES  Sufficient funding is invested in ‘Population Nutrition Promotion’ (estimated from the investments in population promotion of healthy eating and healthy food environments for the prevention of obesity and diet-related NCDs, excluding all one-on-one promotion (primary-care, antenatal services, maternal and child nursing services etc.), food safety, micronutrient deficiencies (e.g. folate fortification and undernutrition)) to create healthy food environments, improved population nutrition, reductions in obesity, diet-related NCDs and their related inequalities. | **FUND1** The ‘population nutrition’ budget, as a proportion of total health spending and/or in relation to the diet-related NCD burden sufficiently contributes to reducing diet-related NCD’s. |
|  | **FUND2** Government funded research is targeted for improving food environments, reducing obesity, NCDs and their related inequalities. |
|  | **FUND3** There is a statutory health promotion agency in place that includes an objective to improve population nutrition with a secure funding stream. |
| DOMAIN 12 – PLATFORMS AND INTERACTION  There are coordination platforms and opportunities for synergies across government departments, levels of government, and other sectors (NGOs, private sector, and academia) such that policies and actions in food and nutrition are coherent, efficient and effective in improving food environments, population nutrition, diet-related NCDs and their related inequalities. | **PLAT1** There are robust coordination mechanisms across departments and levels of government (national, state and local) to ensure policy coherence, alignment, and integration of food, obesity and diet-related NCD prevention policies across governments. |
|  | **PLAT2** There are formal platforms (with clearly defined mandates, roles and structures) for regular interactions between government and the commercial food sector on the implementation of healthy food policies and other related strategies. |
|  | **PLAT3** There are formal platforms (with clearly defined mandates, roles and structures) for regular interactions between government and civil society on the development, implementation and evaluation of healthy food policies and other related strategies. |
|  | **PLAT4** The governments work with a system-based approach with (local and national) organisations/partners/groups to improve the healthiness of food environments at a national level. |
| DOMAIN 13 – HEALTH IN ALL POLICIES  Processes are in place to ensure policy coherence and alignment, and that population health impacts are explicitly considered in the development of government policies. | **HIAP1** There are processes in place to ensure that population nutrition, health outcomes and reducing health inequalities or health impacts in vulnerable populations are considered and prioritised in the development of all government policies relating to food. |
|  | **HIAP2** There are processes e.g. Health Impact Assessment’s (HIAs) to assess and consider health impacts during the development of other non-food policies. |

**Supplementary file 3. Guidance given to experts to determine the level of implementation of policies and infrastructure support**

*[Food-EPI study, the Netherlands, 2019-2020]*

**Introduction to the Food-EPI**

The Food Environment Policy Index (Food-EPI) is developed by the International Network for Food and Obesity / Non-communicable Diseases Research, Monitoring and Action Support (INFORMAS) (https://www.informas.org/modules/public-sector/). The Food-EPI is a tool to assess to what extent government policies and actions for creating healthy food environments have been put forward. The Food-EPI consist of two components: a ‘policy’ component with seven domains on specific aspects of food environments and an ‘infrastructure support’ component with six domains to strengthen systems to prevent obesity and diet-related NCDs (see Figure S2). The 13 domains include 47 good practice indicators.

For each of the 47 Food-EPI indicators, evidence for the existence of national policies in the Netherlands has been extracted from policy documents by the research team. Policies at the national level with a potential influence on the food environment in the Netherlands have been outlined in the “evidence document” under the heading of each of the 47 Food-EPI indicators (see the attachment to the e-mail). This evidence document has been verified by governmental officials.

**Instructions for rating**

You are invited to participate in the Dutch Food-EPI 2020 expert panel. This will involve rating the current level of government policies impacting on the Dutch food environment for 47 good practice indicators, against international best practice, on a Likert scale

from 1 to 5.

The meaning of the Likert scale is:

1:   0-20% implemented compared to international best practice

2:   20-40% implemented compared to international best practice

3:   40-60% implemented compared to international best practice

4:   60-80% implemented compared to international best practice

5:   80-100% implemented compared to international best practice

In rating the degree of implementation of current national policies/actions it is important to assess how these improve the healthiness of food environments. You may think of the following questions:

- What is the scope of the policy/action?
- Which settings, food groups, population groups are included?
- Which type(s) of policy instruments has/have been implemented?
- Are the policies/actions voluntary, mandatory or co-regulatory?
- Do they use a strict nutrient profile model to define unhealthy foods?

There is also a ‘cannot rate’ option, but please only use this if really needed and provide comments in the comment box on why you cannot rate for a particular good practice indicator.

The Food-EPI evidence document  gives you the full details of the current evidence of implementation by the national government in the Netherlands for each good practice indicator.  A summary of the evidence and the international best practice examples (benchmarks) are available within this online questionnaire used for the rating process. **It   is   important   to   read   the   evidence   of implementation and international best practice exemplars (benchmarks) before putting in your rating for each good practice indicator.**

**At the end of each domain we will ask you if you think the government should take more action on one of the indicators in the specific domain. This gives you the opportunity to define specific actions for the government, which will be used to come to a final set of defined and prioritized policy actions for the government.**

The survey will save your ratings automatically online so that you can come back to where you left at a later stage. To send in your final ratings, please click on the right arrow at the bottom of the last page of the survey.

**Supplementary file 4. Prioritization Criteria for Policy and Infrastructure Actions**

*[Food-EPI study, the Netherlands, 2019-2020]*

| **Importance** | **Achievability** | **Equity** |
| --- | --- | --- |
| **Need**  The size of the implementation gap | **Feasibility**  How easy or hard the action is to implement | **Socio-economic effect**  Progressive/regressive effects on reducing food/diet-related inequalities |
| **Impact**  The effectiveness of the action on improving food environments and diets (including reach and effect size) | **Acceptability**  The level of support from key stakeholders including government, the public, public health and industry | **Structures vs. Individuals**  Extent to which a given policy requires environmental change rather than individual choices |
| **Other positive effects** (e.g. on protecting rights of children and consumers) | **Affordability**  The cost of implementing the action |  |
| **Other negative effects** (e.g. regressive effects on household income, infringement of personal liberties). | **Efficiency**  The cost-effectiveness of the action |  |

**Supplementary file 5: Expert panel** *[Food-EPI study, the Netherlands, 2019-2020]*

| Stakeholder  Group | Expertise | Participants approached (n=52) | Participants declined (n=24) | Online benchmarking survey (n=28) | Selection survey (n=17) | Prioritization survey (n=21) |
| --- | --- | --- | --- | --- | --- | --- |
| Academia | Academics in the field of obesity prevention, nutrition and health, food and health policies, medical science, political science, behavioural science (n=12) | 20 | No response=3  No time= 2  Conflicts of interest= 1  Not sufficient knowledge= 2 | 12 | 8 | 10 |
| Health organizations/  health professional associations | Representatives of non-government health professional associations and health organizations in the field of nutrition and health promotion, diet-related chronic diseases (n=6) | 19 | No response=6  No time=5  Not sufficient knowledge= 2 | 6 | 5 | 5 |
| Non-governmental organizations  (NGOs) | Representatives of non-profit organizations in the field of nutrition, health and policies (n=4) | 7 | No response=3 | 4 | 3 | 3 |
| Local governments | Representatives of local governments in the Netherlands (n=6) | 6 |  | 6 | 1 | 3 |

1. Swinburn B, Vandevijvere S, Kraak V, Sacks G, Snowdon W, Hawkes C, Barquera S, Friel S, Kelly B, Kumanyika S, L'Abbé M, Lee A, Lobstein T, Ma J, Macmullan J, Mohan S, Monteiro C, Neal B, Rayner M, Sanders D, Walker C; INFORMAS. Monitoring and benchmarking government policies and actions to improve the healthiness of food environments: a proposed Government Healthy Food Environment Policy Index. Obes Rev. 2013 Oct;14 Suppl 1:24-37. doi: 10.1111/obr.12073. PMID: 24074208. [↑](#footnote-ref-1)
